# Unclasping potentials of genomics and gene editing in chickpea to fight climate change and global hunger threat

**DOI:** 10.3389/fgene.2023.1085024

**Published:** 2023-04-18

**Authors:** Charul Singh, Ramesh Kumar, Hansa Sehgal, Sharmista Bhati, Tripti Singhal, Gayacharan, M. S. Nimmy, Renu Yadav, Santosh Kumar Gupta, Naglaa A. Abdallah, Aladdin Hamwieh, Rajendra Kumar

**Affiliations:** ^1^ USBT, Guru Govind Singh Indraprastha University, Delhi, India; ^2^ Department of Biochemistry, University of Allahabad Prayagraj, Prayagraj, India; ^3^ Department of Biological Sciences, Birla Institute of Technology and Sciences, Pilani, India; ^4^ School of Biotechnology, Gautam Buddha University, Greater Noida, India; ^5^ Division of Genetics, ICAR-Indian Agricultural Research Institute, New Delhi, India; ^6^ Division of Germplasm Evaluation, ICAR- National Bureau of Plant Genetic Resources, New Delhi, India; ^7^ ICAR-National Institute for Plant Biotechnology, New Delhi, India; ^8^ AIOA, Amity University, Noida, India; ^9^ DBT-National Institute of Plant Genome Research, New Delhi, India; ^10^ Faculty of Agriculture, Cairo University, Cairo, Egypt; ^11^ The International Center for Agricultural Research in the Dry Areas (ICARDA), Cairo, Egypt

**Keywords:** chickpea improvement, climate change, CRISPR/Cas-9, genome editing, hunger threat, TALENs, zinc finger nuclease

## Abstract

Genomics and genome editing promise enormous opportunities for crop improvement and elementary research. Precise modification in the specific targeted location of a genome has profited over the unplanned insertional events which are generally accomplished employing unadventurous means of genetic modifications. The advent of new genome editing procedures viz; zinc finger nucleases (ZFNs), homing endonucleases, transcription activator like effector nucleases (TALENs), Base Editors (BEs), and Primer Editors (PEs) enable molecular scientists to modulate gene expressions or create novel genes with high precision and efficiency. However, all these techniques are exorbitant and tedious since their prerequisites are difficult processes that necessitate protein engineering. Contrary to first generation genome modifying methods, CRISPR/Cas9 is simple to construct, and clones can hypothetically target several locations in the genome with different guide RNAs. Following the model of the application in crop with the help of the CRISPR/Cas9 module, various customized Cas9 cassettes have been cast off to advance mark discrimination and diminish random cuts. The present study discusses the progression in genome editing apparatuses, and their applications in chickpea crop development, scientific limitations, and future perspectives for biofortifying cytokinin dehydrogenase, nitrate reductase, superoxide dismutase to induce drought resistance, heat tolerance and higher yield in chickpea to encounter global climate change, hunger and nutritional threats.

## 1 Introduction

Since their origin, land plants have evolved in an essentially hostile environment. These factors deleteriously disturb plant productivity, growth, development and are referred to as stress in plants. Plant stress is due to drastic changes in salinity, temperatures, heavy metals, soil moisture levels, and ultraviolet (UV) emissions. Stresses including both abiotic and biotic are posturing a great menace to agriculture, ecosystems, and noteworthy production losses (Wang et al., 2003; Wani et al., 2016). According to a published report (FAO, 2019), abiotic stress affects roughly 96.5 percent of worldwide rural land areas (Cramer et al., 2011). Crop yields in lower latitude regions are currently declining, whereas yields in higher latitude regions are increasing (Iizumi et al., 2018; IPCC, 2019). Extreme weather occurrences, according to the Intergovernmental Panel on Climate Change (IPCC, 2019), will interrupt and reduce the global food supply resulting in higher food costs. The current estimates of a report by UN reveals that after a continuous decline over a decade, numbers of people suffering from hunger crisis have gradually increased since 2015. Data reveals that at present there are around 690 million people who are hungry which equates to 8.9% of the world population. The report further states that a majority of undernourished population have been found living in Asia and more than 250 million live in Africa, where the numbers are increasing at a very fast rate than anywhere else in the world. On the other hand, there are an estimated 2 billion people who lack access to safe, nutritious and adequate food and are exposed to food insecurity. The report explains that if the present trend persists, the number of people affected by hunger and undernourishment will exceed 840 million, i.e., 9.8% of total population (Arora and Mishra, 2022). The Global Hunger Index (GHI) shows that the number of people who lack regular intake of sufficient calories is increasing. India has ranked poorly for GHI position amongst 107 countries as 100th in 2017, 102nd in 2019, and 94th in 2020. This ranking was counterintuitive considering the fifth rank of India in the world economy. However, Indian policymakers have argued that hunger is an emotional subject and there have been many criticisms and rebuttals of the GHI. Thus, GHI is a misleading hunger index as its methodology ignores genetic factors wherein international norms on stunting and wasting may not be applicable to India (Singh et al., 2021).

During the last two decades, stress has increased by more than two folds, majorly attributed to temperature rise, drought, and salinization of agricultural lands. According to a new meta-analysis study, the worldwide average temperature will rise by almost 5°C by 2,100 (Raftery et al., 2017). Increased heavy metal poisoning of agricultural areas is restricting food output while also posing major health dangers to humans (Rehman et al., 2018). Besides abiotic stresses, biotic stresses also induce stresses through infestations with insects, bacteria, fungi, viruses, and nematodes. Although plants have evolved with various kinds of defence systems to survive, such as halophytes have developed a specific organ to emit salt, as seen by Limonium bicolor’s salt gland (Yuan et al., 2013; 2016). The available basic information on chickpea for the genomic structure (Singh et al., 2013), genetic resources for Dof genes (Yadav et al., 2016), salinity (Mittal et al., 2015), drought (Singh et al., 2012; Singh et al., 2013; Bhardwaj et al., 2014; Mittal et al., 2014; Kumar et al., 2017; Yadav et al., 2019; Bhardwaj et al., 2021), nitrate reductase (Katoch et al., 2016), superoxide dismutase (Singh A. P. et al., 2022) and appropriate strategies (Chandana et al., 2022; Singh R. K. et al., 2022) are necessary and will facilitate the deployment of biotechnological approaches to develop heritably engineered transgenic chickpea plants with upgraded stress resistance. To combat food scarcity, an amalgamation of outdated plant breeding and novel methodologies such as molecular plant breeding and gene editing must be applied. Targeted genome editing boosted grain size related metrics viz; the number of tillers, and protein quality in rice and corn including several monocots and dicots (Shan et al., 2014; Sedeek et al., 2019). The introgression of quantitative trait loci (QTLs) genomic regions implicated for stress tolerance resulted in the introduction and/or over expression of selected genes into genetically altered plants and appear to be a promising alternative for hastening the breeding of “better” crop plants including chickpea. Thus, genetic engineering, often known as genetically modified (GM) crop technology allows scientists to transfer valuable genes from a completely separate gene pool into the crop plants with the least amount of disturbance to the plant genome and is frequently advocated as an answer for raising yields in crops including chickpea around the world, predominantly in under-developed areas where food insecurity and low crop production are major concerns (Nelson et al., 2007).

Chickpea a member of the fabacean family, one of the extremely significant and second largest leguminous food crops across the globe, has an extraordinary mandate due to the high dietary value of the grain. Today, chickpea ranks third among leguminous food plants for global production, behind field pea (*Pisum sativum L*) and beans (*Phaseolus* spp.) (FAO, 2019). It is cultivated in more than 55 countries across the globe on an estimated 14.56 million hectares area generating 14.78 million tons of total production. Chickpea production, on the other hand, is insufficient to supply the protein requirements of an ever-increasing human population (Reddy et al., 2016; Henchion et al., 2017). A foremost task for crop breeders is enhancing crop production to feed probably ∼10 billion worldwide civilization by 2050 (Hickey et al., 2019). Among legumes family members, after common bean, Chickpea is the economically as well as nutritionally important crop plant. However, cultivation of chickpea is limited due to the various abiotic and biotic stress factors. Being rabi crop, it also faces low temperature stress especially during reproductive stage leading to significant loss in its production. Recently, a detailed review focusing on impact of various stresses on chickpea showed how slightest change in condition can alter the development of the plant (Rani et al., 2020; Akinlade et al., 2022). Thus, various strategies have been applied to improve the tolerance of chickpea employing various conventional breeding techniques but time consuming and laborious processes are the challenges faced by breeders in developing a cultivar tolerant to stresses (Jha et al., 2014).

However, genome editing technologies have tremendous effects on plant breeding techniques to guard crop plants against numerous tasks and augment crop yield (Taranto et al., 2018). Editing the target DNA sequence by adding, selecting, or substituting nucleotide bases is a cutting-edge molecular biology technique. The techniques such as ZFNs, TALENs, Base Editors, CRISPR/Cas9, and Primer Editors are currently being used for genome editing. The CRISPR/Cas9 technologies corroborate the utmost operational GE machineries since these are precise, less expensive, speedy, and consent for numerous site-specific genome editing (Zhu et al., 2017). Hence, in this review article, we are focusing on genetic engineering approaches as comprehensive efforts for biofortifying cytokinin dehydrogenase, nitrate reductase, superoxide dismutase to induce drought resistance, heat tolerance and higher yielding diversities that will upsurge chickpea productivity, usefulness for chickpea growing farmers to encounter global climate change, hunger and nutritional threats.

## 2 Bottlenecks in chickpea gene editing applications

Presently, India is the world largest producer of chickpea (Khine et al., 2022). Yet we dawdle behind other chickpea growing countries in productivity. Hence, it is important to improve the productivity of chickpea. To sustain chickpea production development of climate resilient cultivars are needed. Scientific community around the globe had put lots of effort to enhance yield of chickpea still not able to reach at significant level. The primary reason is that chickpeas have inherently narrow genetic base as they have been extorted to natural selection, domestication syndrome, founder effect, etc. (Abbo et al., 2003).

Chickpea transformation which was accomplished using cutting-edge biotechnological techniques, is a crucial part for genetic enhancement and a prerequisite for genome editing. The efficient production of transgenic chickpeas is hampered by tissue cultures that refuse to cooperate and the occasional chimerism that is found during transformation. Legumes including chickpea are well known to be both resistant to the uptake and integration of introduced DNA (Yadav et al., 2017) and recalcitrant in terms of regeneration (Ochattet al., 2018). Being recalcitrant in nature chickpea transformation is difficult and a robust transformation method is a prerequisite for researchers to carry out the genetic transformation studies in the crop. Although several labs have reported chickpea transformation, the limitations associated with the reproducibility of the technique (Huda et al., 2000; Das Bhowmik et al., 2019), poor *in vitro* rooting (Polowick et al., 2004), low transformation efficiency, regeneration capacity and non-transmission of genes to subsequent generations (Sarmah et al., 2004) remain problematic. The reports available till date indicate majority of the chickpea transformation works have been carried out using *Bacillus thuringiensis* genes for pod borer resistance (Das et al., 2017). However, recently an agrobacterium mediated transformation system in six cultivars of chickpea with 8.6% efficiency has been established (Sadhu et al., 2022). Further, major factors leading to narrowing of genetic base, utilization of available genetic resources for devising strategies to broadening the genetic base, facilitating the transformation strategies and also provide opportunities for genome editing applications in chickpea have been explained (Singh A. P. et al., 2022).

Chimerism is another challenge due to which recovery of stable transgenic lines decline. For instance, earlier researchers have revealed that the percentages of non-transmitting, chimeric lines in chickpea and lentil, were 22% and 29% respectively (Christou, 1990; Dillen et al., 1997; Sarmah et al., 2004; Celikkol Akcay et al., 2009). The effectiveness of recovering stable transgenic lines is decreased by the presence of chimeric tissues (Christou, 1990; Dillen et al., 1997; Sarmah et al., 2004; Celikkol Akcay et al., 2009). Measures for removing chimerism in legumes including chickpea yet have not been published except a single report on lentil (Celikkol Akcay et al., 2009), which showed reduced chimerism and stable expression of a GUS reporter in successive generations.

The carotenoid biosynthesis candidate genes have been identified as a knockout target to increase the carotenoid concentration in chickpea (Rezaei et al., 2016). Developing different abiotic stress tolerant lines would be the future genome editing target in case of chickpea. First report of CRISPR/Cas9-mediated editing of chickpea protoplasts was recently published. Scientists from Australia’s Royal Melbourne Institute of Technology (RMIT) have demonstrated the feasibility of gene editing in chickpea laying a technical foundation for future trait discovery and improvement by creating knockouts of 4-coumarate ligase (4CL) and Reveille 7 (RVE7) genes associated with drought tolerance in chickpea (Badhan et al., 2021). Thus, it is evident that though genome editing is progressing well, the recalcitrant nature of the crop for *in vitro* gene transfer and regeneration is a major challenge and successful chickpea traits improvement will remain dependent on the efficient plant transformation and regeneration protocols.

## 3 Genome editing (GE) tools and strategies

GE technologies have been continuously in use for dissimilar plants including species such as *Arabidopsis* and major crops such as rice, maize, wheat, and economically less important crops such as strawberries and peanuts. In the majority of the cases, these techniques have been employed for fundamental research as proof-of-concept or to examine gene functions. Several market-oriented qualities such as improved agronomic properties, upgraded quality of food and feed, higher endurance to abiotic and biotic stresses and herbicide tolerance have been addressed. The traditional genetic engineering strategies have several flaws and limitations, one of which is the difficulty of manipulating big genomes in higher plants (Nemudryi et al., 2014). The development of revolutionary tools for procreation and biotechnology, a genetic engineering application area, has attracted a lot of attention, resulting in the rapid development of valuable tools. Genetic modification for targeted gene augmentation is widely used in the field of plant science for both fundamental research and the development of desirable characteristics in commercial crops.

The generations of GM crops rely on randomly inserting new stretches of DNA sequences into the genome. The inserted genome may affect or inactivate other neighbouring genes’ activity which is one of the major concerns of this strategy. However, genome editing makes advantage of more contemporary knowledge and technology to allow for the alteration of a definite area of the genome, enhancing the preciseness of the insertion, avoiding cell death, and providing flawless duplication (Voytas, 2013; Voytas and Gao, 2014). Genome editing, also known as genome engineering, is one of the utmost talented machineries applied in biological investigation (Hu et. al., 2008), engineering revolutions and right now a sophisticated tool that allows for precise changes to the genome, using only some of the nucleotides in a living cell’s genome sequence. Despite those facilitations, various obstacles exist which include public scepticism about GM crops, which is heightened when “foreign” genes from remotely related creatures are introduced, as this is viewed as “unnatural,” despite mounting shreds of evidences to the differences as natural sweet potato variations are well recognized to include T-DNA from the bacteria *Agrobacterium tumefaciens* and can be seen as “natural GMO” (Rastogi Verma, 2013; Lucht, 2015). GM crop production is costly, and the biosafety education required to come across controlling criteria adds significantly to the cost, which is predicted over $120 million per trait (Lusser et al., 2012). As a result, GM technology could not be utilized to its potential, except in a few crops by a few countries. Similarly, in chickpeas very limited efforts on transformation for a few selected target traits have been accomplished (Table 1). Monitoring of necessities also cause significant delays in product introductions. Targeting gene expression with homologous recombination is a valuable way for obtaining facts on genetic expressions (Capecchi, 2005; Gaj et al., 2013). However, the technique’s implementation has been limited owing to its low efficiency, extended study duration, mutagenesis consequences, and off-target impacts. Here, various approaches have been discussed that are/may be used in chickpea genome editing like site-specific recombinase or Site-Specific Nucleases that could be used to modify the genome.

**TABLE 1:** Genetic transformation of Chickpea.

Genotype	Explant	Transgene	Promoter	Gene delivery system	Aim	References
C 235, BG 256, Pusa 362 and Pusa 372	Cotyledonary node	*cry1Ac*	*CaMV35S*	Agrobacterium-mediated	Insect resistance against *H. armigera*	Sanyal et al. (2005)
ICCC37	Epicotyl	*cryIAc*	*CaMV35S*	Agrobacterium-mediated	Insect resistance against *H. armigera*	Indurker et al. (2010)
Annigeri	Cotyledonary node	*P5CS*	*CaMV35S*	Agrobacterium-mediated	Salinity tolerance	Ghanti et al. (2011)
P-362	Cotyledonary node	*cry1Ab* and *cry1Ac*	*CaMV35S* and synthetic constitutive expression promoter (*Pcec*)	Agrobacterium-mediated	Insect resistance	Mehrotra et al. (2011)
DCP 92–3	Embryonic axis	*cry1Ab/cry1Ac*	Rice *actin1* and soybean *msg*	Agrobacterium-mediated	Insect resistance	Ganguly et al. (2014)
Gokce	Mature embryo	miR408	CaMV35S	Agrobacterium-mediated	Drought tolerance	Hajyzadeh et al. (2015)
ICCV 89,314	Single cotyledon with half embryo	*cry1Ac*	*RuBisCO* small subunit and ubiquitin	Agrobacterium-mediated	Insect resistance to target *H. armigera*	Chakraborty et al. (2016)
DCP 92–3	Axillary meristem	*cry1Aabc*	*CaMV35S*	Agrobacterium-mediated	Insect resistance	Das et al. (2017)
PBA HatTrick	Half-embryonic axis	nicotianamine synthase 2 and ferritin	*CaMV35S* and nopaline synthase	Agrobacterium-mediated	Iron biofortifcation	Tan et al. (2018)

### 3.1 GE through site-specific recombinase (SSR): A molecular machine for genetic reformation

SSR is a frequently used genetic engineering technique for permanently altering the target genome. Lots of site-specific recombinase systems have been developed to accomplish DNA reorganizations including Cre/loxP and Flp/FRT(Araki et al., 1995; Allen and Weeks, 2005; Allen et al., 2009; Wang et al., 2011). SSRs can be used to manipulate genomes and stimulate or deactivate gene expression in numerous organisms (Wang et al., 2011). Recombinase has been widely utilized to modify the DNA of mammals, yeast, plants, and bacteria by introducing knockout or knock-in mutations into their genomes (Abdallah et al., 2015). One of the benefits of recombinases is that they are not reliant on intracellular repair mechanisms (Abdallah et al., 2015).

**FIGURE 1:**
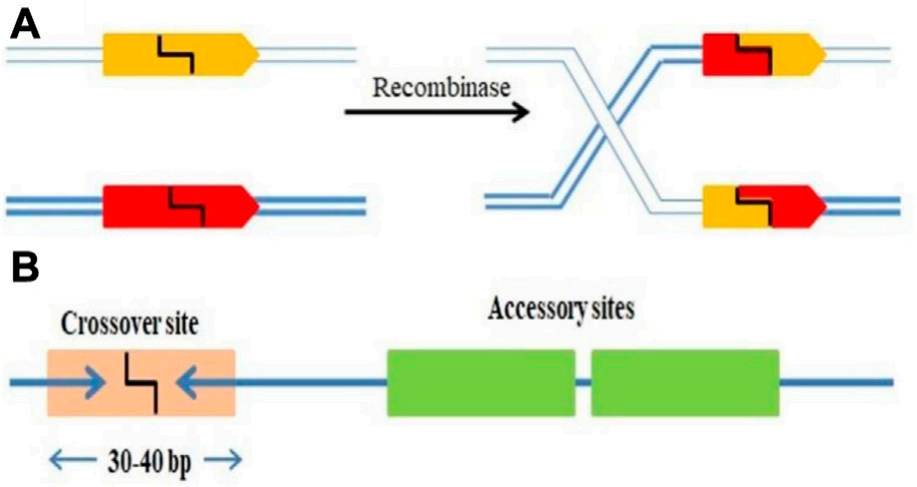
**(A)** SSRs facilitating DNA strand breakage at two points (pointed boxes) followed by rearranging (“swap”) the broken ends and reconnecting them in the new configuration. **(B)** A crossover site (light orange box) with inverted repeat symmetry (two blue arrows) binding an SSR dimer and containing the broken bonds and re-joined by the SSR at its centre (typically 30–40 bp). The crossover site could have accessory sites (Green boxes) that bind more SSR and/or regulatory protein subunits.

SSRs are molecular machines that allow DNA molecules to be cut, paste and editing by adding, removing, or inverting precisely defined DNA segments (Grindley et al., 2006; Gaj et al., 2014). The mechanism incorporates and eliminates the bacteriophage DNA from a definite location in its host genome. *Escherichia coli* was the first example of site-specific recombination in bacteria (Landy, 2015). Each strand of recombining DNA has two core-type sites, which are inverted repeat recombinase binding sites, that flank an identical 7 bp “overlap region” called as O in both DNAs (Rutkai et al., 2006). The two active Ints on one side of the “overlap region” cleave and interchange the top strands of the DNA to form a four-way DNA junction called Holliday junction (HJ), which is subsequently resolved to recombinant products by the other pair of Ints cleaving and trading the bottom strands of the overlap region. Additional DNA sequences that encode binding sites for the second (NTD) DNA binding domain of Int and the accessory DNA bending proteins, IHF, Xis, and Fis are added to two of the four core-type sites. However, some sites are considered necessary either only for excisive recombination between the attL and attR sites, or for integrative recombination between attP (on the phage chromosome) and attB (on the bacterial chromosome), or needed for both reactions (Landy, 2015). Two short DNA sequences are brought together at different positions inside one DNA or in distinct molecules; the DNA fragments are damaged at specified phosphodiester links inside DNA, and the damaged ends are re-joined in a new configuration to generate recombinants (Figure 1A). The identification of sequence and biochemical catalytic phases of this procedure is carried out by the site-specific recombinase, a system-specific enzyme. It is frequently discussed as conventional particular recombination to discriminate it from procedures like homologous recombination (HR), transposition, and non-homologous end-joining, since it does not need DNA synthesis, fragmentation, or cofactors. In more complicated systems, the SSR dimer’s “crossover site” neighbouring to “accessory” sequences is acknowledged and assured by the SSR and/or additional proteins (Figure 1B).

The first and foremost application, which was established more than two decades before, is the elimination of a targeted gene from a locus (Dale and Ow, 1991; Russell et al., 1992), which has been monitored quickly by site-specific integration (SSI) to construct accurate one-copy transgene loci and determining complex loci to one copy (De Buck et al., 2007; Srivastava and Ow, 2015). These applications were established for the first time using the Cre-lox system and then lengthened to include additional SSR systems such as Par A, FLP-FRT, phiC31, R-RS, Cin H, and Bxb1 (Sugita et al., 2000; Li Z. et al., 2009; Moon et al., 2011; Thomson et al., 2012). SSR systems can knock down the genome liable on the positioning of the definite sites adjoining the target site. These systems are applicable in numerous plant species and can be used in chickpeas for genetic modification tasks: 1) marker gene elimination and 2) particular external gene insertion *via* site-specific integration.

#### 3.1.1 Basic steps involved in site-specific recombination systems

The three SSR systems identified in the initial 1990s, namely, Cre-lox from bacteriophage P1 of *E. coli*, FLP-FRT from *Saccharomyces cerevisiae*, and R-RS from *Zygosaccharomyces rouxii* are still in use for incorporating diversities in crop plants and can be employed also in chickpea in order to encounter the global climate change and hunger threat. Enzyme recombinase Cre, FLP, or R catalyses recombination in between its analogous recombination sites lox, FRT, or RS, in these recombination systems. Each recombination target site (RTS) up to 34 bp in length has an unequal core/spacer section flanks inverted repeats (RE and LE) that act as recombinase binding locations. Several regions confer the cross over sites, while their unevenness provides the recombination site directions. The reaction steps are 1) identification along with the binding of recombinase dimers to mandatory sites, 2) synaptic complex formation between bound positions, 3) strand exchange and fusion proceedings mediated by recombinase, 4) synaptic complex segregation (Whiteson and Rice, 2008). Some other SSR systems for plant transformation developed in recent years, such as the ΦC31- att and λ-att systems, which consist of a recombinase protein, phiC31 or λ integrase (Int), and catalyse recombination between unrelated recombination sites identified as attB and attP to produce fusion sites attL and attR. The translocation, co-integration, inversion, and deletion can occur subject to the location of attB and attP. However, to catalyze the reverse reaction and reproduce attL and attR hybrid sites from attB and attP, a supplementary excision/resolvase protein is required; therefore, the incorporation reaction is one way in absence of protein.

#### 3.1.2 Site-specific recombinase families

System-specific SSR organization unfolds that the majority of the thousands known site-specific recombination systems are divided into 2 families. These are recombinases of serine and tyrosine, termed after the identification of amino acid residues at the nucleophilic active sites. The side chain of serine or tyrosine breaks a strand by attacking the phosphodiester bond of the DNA and covalently links at the damaged DNA strand end. The phosphodiester link between the DNA preserves bond energy, allowing recombinant strands to be re-joined without the use of cofactors like ATP or additional polymerase or ligase processing. The common features between these two families are crossover site recognition by SSR dimer and catalysis within the SSR tetramer, although their mode of action is different and the proteins have no sequence and organizational similarities (Castillo et al., 2017).

**FIGURE 2:**
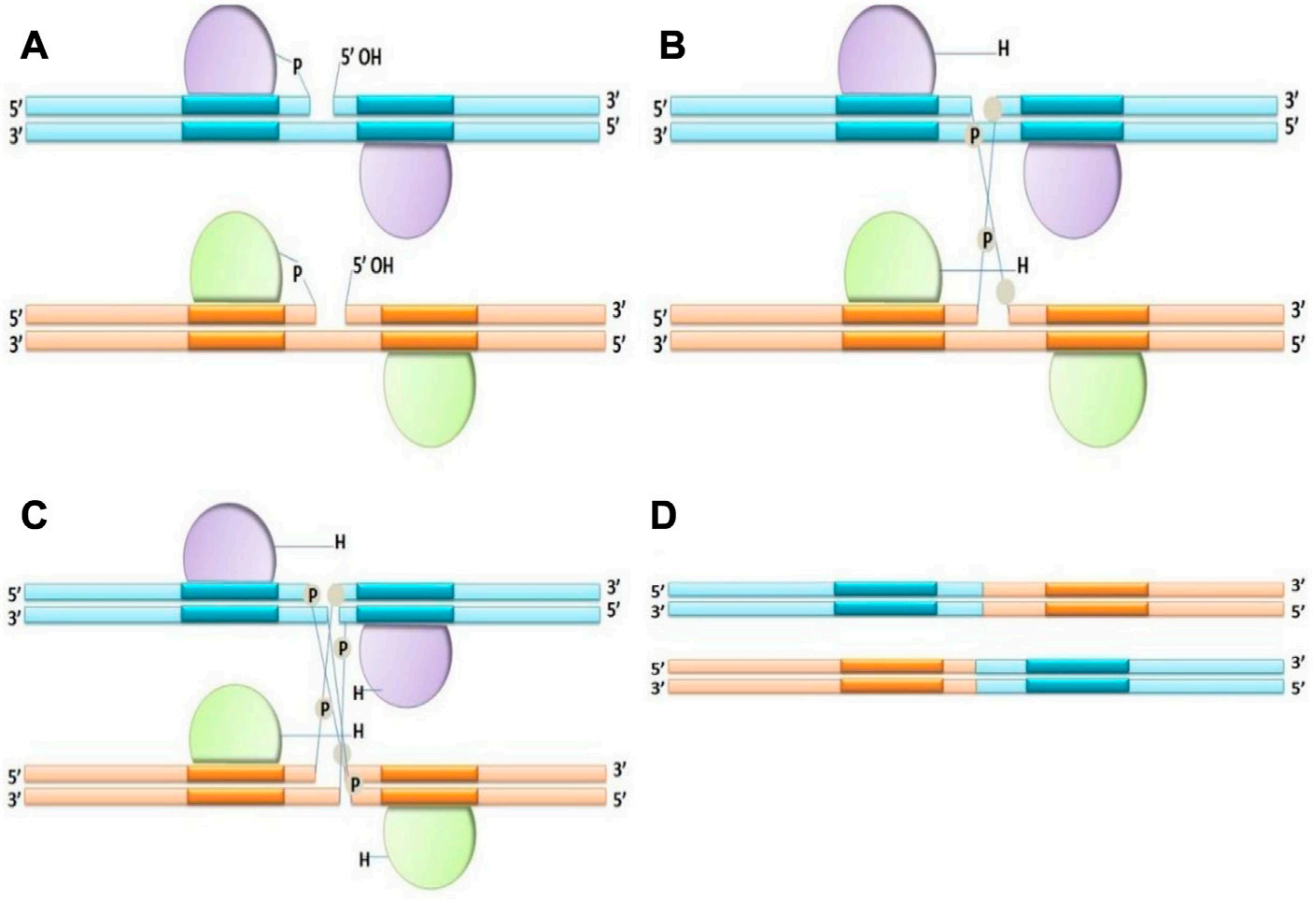
Mechanism of Tyrosine recombinase **(A–D)**, making an intermediate Holliday junction by expurgating and interchanging one pair of DNA strands, followed by cutting and interchanging the other couple of strands.

**FIGURE 3:**
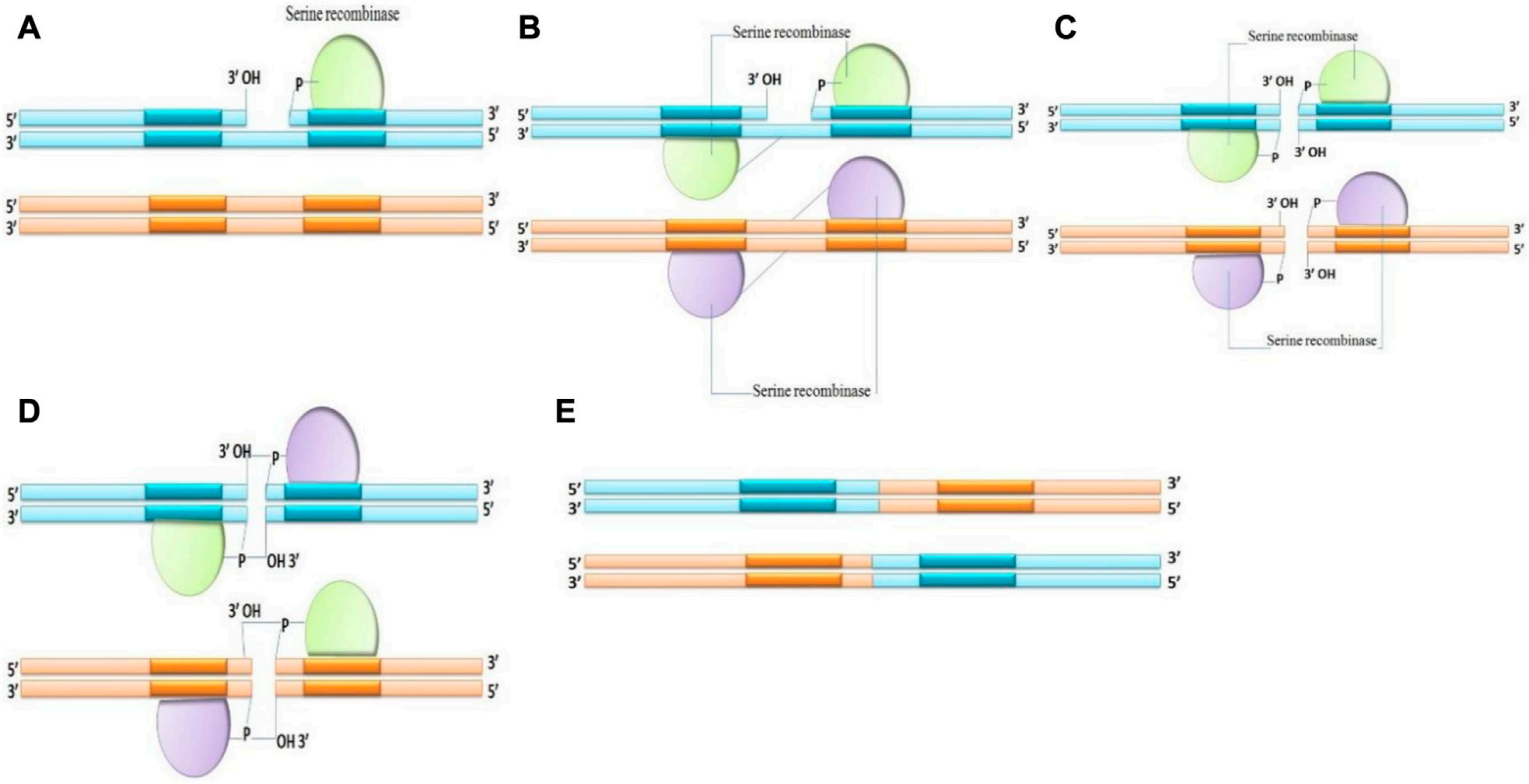
Mechanism of Serine recombinase **(A–E)** making double-strand breaks in all crossover sites before reshuffling the fragmented DNA ends and rejoining the strands.

1. ***Tyrosine recombinases:*** Tyrosine side chain attacks a specific phosphodiester bond in the recombination site. When tyrosine recombinase attacks the DNA strands, the hydroxyl group of tyrosine residue covalently bonds to each 3′end of the damaged DNA. Tyrosine recombinases interchange, disrupt and rejoin two DNA strands at once; their reactions continue through a “Holiday” or 4-way connection intermediary, in which 2 strands are non-recombinant while the remaining 2 are recombinant (Figure 2). In experimental genetics and biotechnology, several tyrosine recombinases have been utilized; in fact, the most extensively used SSRs such as Cre (Sauer and Henderson, 1989) and FLP (Golic and Lindquist, 1989) and R (Onouchi et al., 1991) are members of this family.
2. ***Serine recombinases:*** Serine recombinase breaks the DNA strand by the aggression of the phosphodiester with the OH group of serine amino acid and covalently attaches the recombinant DNA to the 5′end at the breakdown. During recombination, serine recombinases create instantaneous double strand breaks in both recombining sites and there is no Holliday junction. A unique subunit rotation mechanism causes recombination by swapping the locations of the cut DNA ends (Figure 3). The upper and lower strand breaks are always 2 bp apart and proportionally positioned in the midpoint of the crossover sites (Smith and Thorpe, 2002; Marshall Stark, 2015). The serine recombinases family contains phiC31 Integrase and phi C31 excisionase (Thorpe and Smith, 1998).

#### 3.1.3 Applications of site-specific recombination systems

The advantages of using SSR over other methods for DNA rearrangement are concise due to its specificity, efficiency, and simplicity as SSR is rigorously restricted to a particular DNA sequence consisting of a site of 30–40 amino acids (Gaj et al., 2013; Carroll, 2014). *In vitro* and *in vivo*, site-specific recombination could be exceedingly quick and effectual under optimum conditions (Nash et al., 1996). The SSR encourages full recombination by breaking and re-joining of all 4 DNA filaments at the recombination sites. There are no additional cofactors required (Olorunniji et al., 2016).

The first application of Cre to catalyze the exclusion of selectable marker genes from transgenic tobacco (Dale and Ow, 1991; Albert et al., 1995) happened in the early 90s followed by several other reports such as in rice (Hoa et al., 2002; Radhakrishnan and Srivastava, 2005; Hu et al., 2008), wheat (Srivastava et al., 1999), tomato (Stuurman et al., 1996), barley (Kapusi et al., 2012), soybean (Li et al., 2009), Arabidopsis (Vergunst and Hooykaas, 1998), maize (Zhang et al., 2003; Kerbach et al., 2005; Anand et al., 2019) and in Chickpea-Rhizobium Rcd301 utilizing site-specific homologous recombination, the hup gene fragment from cosmid pHU52 was incorporated into the genome followed by addition of two fragments of the strain Rcd301’s own genomic DNA to flank the cloned hup genes for successful integration (Vijaya Bhanu et al., 1994).

### 3.2 GE through oligonucleotide directed mutagenesis (ODM)

ODM, which dates back to the early 1980s, is a gene editing tool that is a base pair specific, precise and non-transgenic that has been greatly advanced to create unique and commercially relevant features in agriculturally important crops and can also be employed in chickpea. ODM, after its successful application in mammalian systems, has set off as an alternative novel gene edition method for plants (Abdurakhmonov, 2016; Sauer et al., 2016). ODM is a technique for targeted mutagenesis that employs a 20–100 base oligo nucleotide whose sequence is alike to the target sequence in the genome excluding a unit base pair change to achieve site-specified editing of the sequence of interest (Rádi et al., 2021). When these short oligonucleotide sequences are temporarily exposed to cultured plant cells, the repair template matches and binds to the homologous plant DNA sequence. The cell’s inherent repair mechanism recognizes the single base mismatch between its DNA and the repair template once it has been attached. The cell will restore its DNA sequence by replicating the discrepancy in its DNA sequence. As a result, the oligo nucleotide is destroyed by the cell, and the required particular alteration in the plant’s DNA is created. Plants with the precise mutation are then regenerated using tissue culture techniques, and standard breeding techniques are used to efficiently breed the desired features into elite plant varieties while removing undesired characteristics.

ODM has been greatly advanced using Rapid Trait Development System (RTDS). The RTDS™ machinery deals with a quick, explicit, and non-transgenic breeding substitute for traits enhancement to create unique commercially relevant features in agriculturally essential crops (Gocal et al., 2015). The RTDS method uses the cell’s regular DNA repair system to alter particularly targeted bases in the genome for utilization of chemically generated oligo nucleotides. These oligo nucleotides serve as restoration templates causing DNA mismatches at the target location.

#### 3.2.1 Applications of ODM technology

The ODM approach has been used successfully in a variety of plant crops, including herbicide tolerance (Zhu et al., 1999; Okuzaki and Toriyama, 2004; Dong et al., 2006; Rádi et al., 2021). Single point mutations are one of the ways of ODM applications in plants to transform endogenous loci(s) by targeting Aceto-Hydroxy Acid Synthase (AHAS) gene. Herbicides that block this enzyme such as imidazolinones (Imis), chlorsulfuron (CS), pyrimidinyl thiobenzoates, sulfonylureas (SUs), and bispyribacsodium (BS) make mutant enzymes easily selective (Tan et al., 2005). HRAC Group B and Australian Group B herbicides are classified as Group 2 herbicides in the Canadian herbicide classification system. One of three amino acid sites P197, S653, and W574 were targeted based on numbering on the Arabidopsis AHAS protein sequence to accomplish struggles to the afore mentioned herbicide chemistries. The study defining the fruitful applications of ODM was first conducted in the tobacco Nt-1 cell suspensions (Beetham et al., 1999; Ruiter et al., 2003), henceforth on maize (Zhu et al., 1999; Zhu et al., 2000). Other crops such as rice (Okuzaki and Toriyama, 2004), rapeseed (Ruiter et al., 2003; Gocal et al., 2015), including Arabidopsis (Kochevenko and Willmitzer, 2003) were also studied and tested. The transformation rates are liable to the crop, its cellular biology, the type of oligonucleotide and its concentration, the strand being directed, and the specific mutation taking place, which makes it difficult to compare different oligonucleotide delivery systems. In many aspects, the application of a fluorescence conversion approach, in which a BFP that is a Blue Fluorescent Protein could be converted into green fluorescent protein (GFP) just by editing a unit nucleotide of the Blue Fluorescent Protein gene, has improved oligo nucleotide mediated conversions. For example, oligo nucleotide length optimization and end protective chemistries have shown the potentials in boosting conversion rates (Sauer et al., 2016).

The protoplasts, generated through a BFP transgenic strain, were evaluated for the BFP to GFP gene edit for demonstrating the efficiency of oligo nucleotide mediated conversions in Arabidopsis. The findings show that oligo nucleotide mediated conversions have an excellent way to induce precise alterations in Arabidopsis. Moreover, these oligo nucleotide optimizations can have a big impact on the frequency of targeted modifications (Sauer et al., 2016). Furthermore, ODM has the potentials to improve crops without introducing additional genetic material by utilizing the plant’s genome to boost abiotic (heat, drought, salinity) and biotic disease resistance (insect, bacterial, and virus), nutritional value, as well as its yield. ODM is presented as one of the numerous innovative breeding approaches that have set about the commercialization of food plants due to its capacity to accurately change sequences in genomes. Some commercial crops have been exploited *via* ODM such as maize, wheat, rice and rapeseed for herbicide tolerance as mentioned above. In 2016, A US based company Cibus put forward a herbicide tolerant rapeseed in several EU countries as a test case by using ODM in Rapid Trait Development System (RTDS) (Fladung, 2016). So far, no work has been reported in chickpea using ODM. Nevertheless, it is equally applicable in chickpea as well and may be expected to be done in near future.

### 3.3 GE mediated through site specific nucleases (SSNs)

Sequence-specific nuclease-based mutagenesis was first employed in plant research 15 years ago in 2006 (Razzaq et al., 2019) where engineered nucleases (ENs) were primarily used (Bruce Wallace et al., 1981) and engineered nucleases are divided into four categories: Zinc-Finger Nucleases (ZFNs), Transcription Activator Like Effector Nucleases (TALENs), Mega-nucleases and Clustered Regularly Interspaced Short Palindromic Repeats (CRISPR)-Systems. SSNs work by building endonucleases that can cleave DNA onto a specific sequence in the genome. SSNs can have DNA or RNA binding pockets that attach to specific target sequences (Gaj et al., 2013; Carroll, 2014). These evolving technologies are progressing at breakneck speed, particularly in the realm of CRISPR-based genome editing (Abdallah et al., 2015; Kamburova et al., 2017) and are equally applicable in food legumes including chickpea.

#### 3.3.1 GE mediated through zinc finger nucleases (ZFNs)

**FIGURE 4:**
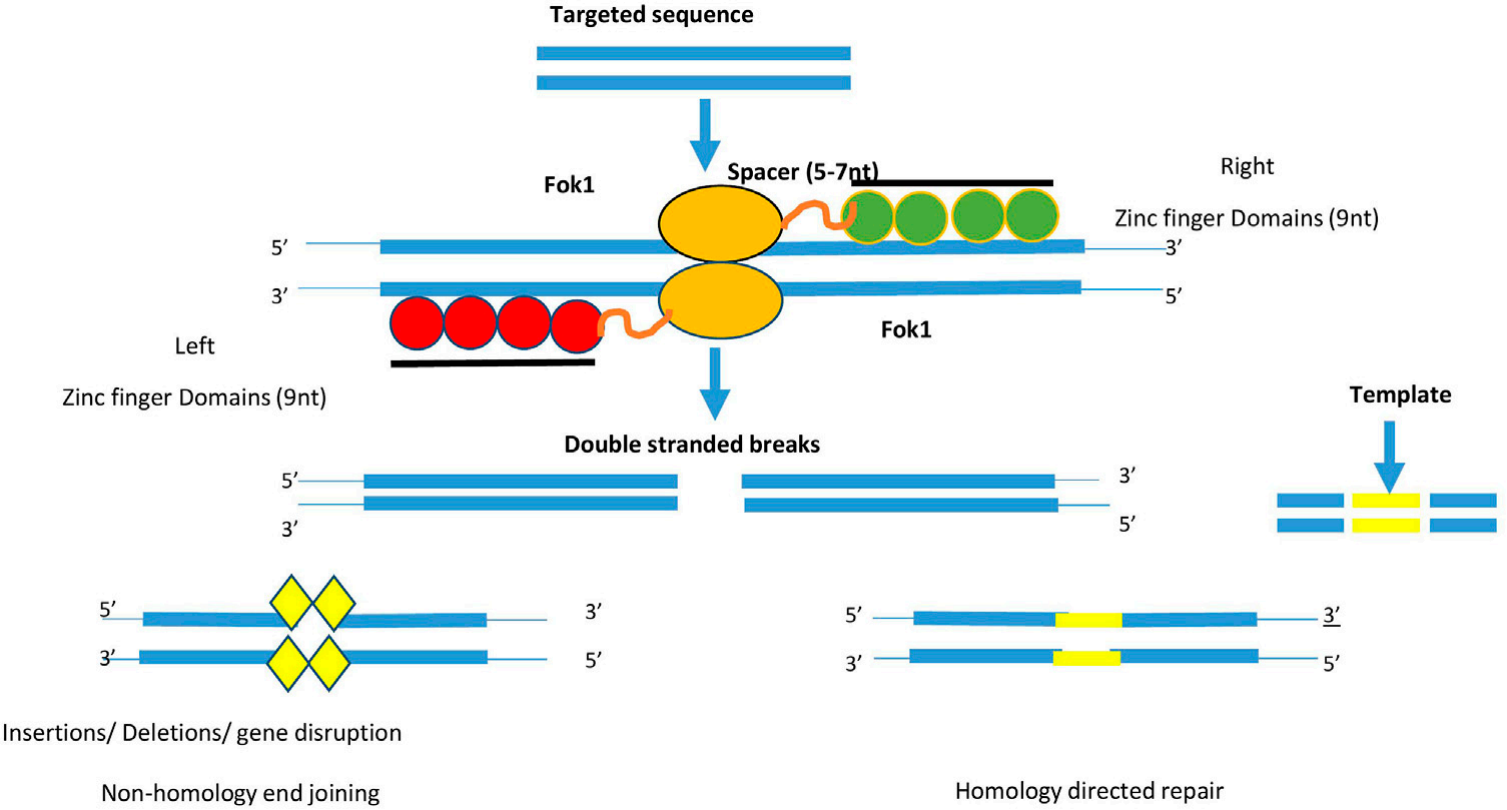
Diagram depicts the components required for the action of ZFN. It consists of 4–6 zinc finger domain (green and red colour) which binds to the targeted DNA sequence. For the action of FoI enzyme, type II restriction enzyme (yellow colour). Two monomeric sequence attaches on DNA sequence and allow FokI to create double stranded breaks. These breaks will be repaired *via* non-homology end joining or Homology directed repair method.

Currently, scientists have access to several techniques that can assist them to tackle difficulties related to precise genome editing in plants. Kim et al. (1996) discovered for the first time that protein domains like “zinc fingers” combine with FokI endonuclease domains, which act as site-responsive ZFNs and cleave DNA *in vitro* in well-defined locations (Miller et al., 2007). The chimeric protein has a modular structure because each one “zinc finger” domain recognizes nucleotides in the form of a triplet. This approach was used to alter cultured cells including both model and non-model plants (Cai et al., 2014). These were the first class of proteins to target a specific region of DNA and make double-stranded breaks. For their action *Flavobacterium okeanokoites I* (Fok1) nuclease enzymes assist them (Khandagale and Nadaf, 2016). The Cys2His2 type Zinc fingers are considered as most common eukaryotic transcription factors, whereas zinc finger nucleases are engineered restriction enzymes. It comprises 30 amino acids present in ββα fold and the inking of zinc provides more stability to the structure (Chen et al., 2014). The crystalline form of Zinc finger protein showed that it binds to major grooves of target DNA (Aslam et al., 2019). Structurally, its monomer consists of two important domains, namely, the DNA binding domain and DNA cleavage domain or nuclease domain. Out of an array of 4-6, zinc finger domains each of them recognizes 3bp of DNA sequence as shown in Figure 4. Using the phage display method wide range of ZFNs domains recognizing specific DNA triplets are identified. Knowing distinct domain recognized by ZFNs allow us to fuse them in tandem *via* linker peptide to form polydactyly zinc finger proteins that can target a wide range of DNA sequences (Gaj et al., 2016). Recent studies have tried to include more fingers to recognize longer and cleave rare targets (Urnov et al., 2010). The specificity of adherence to DNA is influenced by interaction with adjacent domains too (Petolino, 2015). For high specificity two ZFN monomers are required as the FokI nuclease domain act in dimerized form. Furthermore, the amino acids positioned at first, second, third, and +6 at the starting of the zinc finger alpha helix, contribute to peculiar binding to sites (Osakabe and Osakabe, 2015).

To reduce off-site cleavage, FoKI variants have been developed which require heterodimerization between two monomers of ZFN (Ran et al., 2018). Engineering methods are widely used for the construction of engineered ZFNs, identification of triplet sequences, modular assembly, and oligomerized pool. The drawback of this approach is that ZFNs can bind to neighbouring fingers as well as to bases present outside of the proximity of the targeted DNA triplet (Urnov et al., 2010). GE through ZFNs yields modification with efficiencies of more than 10% by creating double-stranded breaks (Miller et al., 2007). The efficiency of mutagenesis was reported in Arabidopsis and it was found to be 78% in case of simple deletions, 13% in simple insertions, and approximately 8% in deletions with long insertions (Lloyd et al., 2005). In another study, the constitutive expression of ZFN resulted in a 2% mutation and deletion of sequence ranging from 1 to 80 bp (de Pater et al., 2009).

##### 3.3.1.1 Application of ZFN technology

Despite of challenges faced during the construction of ZFNs, they have been widely used to modify genes of cultivated crops *Arabidopsis*, tobacco, maize, soybean, and canola (Mushtaq et al., 2019). In maize disruption of endogenous inositol phosphatase kinase 1 gene by the introduction of PAT gene cassettes lead to the development of herbicide-tolerant cultivars and simultaneously alteration in inositol phosphate of developing seeds (Zhang et al., 2018). In another approach, ABA INSENSITIVE-4 (ABI4) gene was mutagenized in *Arabidopsis*, and the frequency of insertion and deletion was a maximum of 3% in nine transgenic lines. However, when estrogen-inducible ZFNs were used to create mutations in *Arabidopsis,* in the first generation the rate of mutations was 7% and 16% in the two genes, namely, alcohol dehydrogenase1 and transparent test4 (Zhang F. et al., 2010). In the oil seed family, ZFN was performed in soybean and brassica to improve agronomic traits. A similar approach was made to create mutations in dicer-like (DCL) genes in soybean to develop the Zinger finger consortium by context-dependent assembly (Curtin et al., 2013). In *Brassica napus*, the method has been used for activation of β-ketoac- ACP synthase II, resulting in a decrease in the production of palmitic acid and entire saturated fatty acid content (Gupta et al., 2012). Recently, purified ZFN monomer proteins were isolated from bacterial cultures and delivered into unmodified microspores to edit the inositol pentakiphosphatase kinase1 gene, which is found to be involved in catalysing the end step of phytic acid production (Bilichak et al., 2020). In the populous, the heat-inducible ZFN system mutagenizes floral genes at a rate of 0.3% (Lu et al., 2016). In tobacco, mutations were targeted in SuRA and SuRB conferring herbicidal resistance to imidazolinone and sulfonylurea compounds (Maeder et al., 2008; Townsend et al., 2009). ZFN approach can be used to facilitate multiple knockouts of the gene as seen in wheat, three homologous copies of the acetohydroxy acid synthase gene were targeted simultaneously (Ran et al., 2018). Against biotic stress, plants develop resistance against the pathogen, and ZFNs were artificially designed to bind against the circular single-stranded DNA of begomovirus (Chen et al., 2014). Earlier, an artificial zinc finger protein (AFP) without a nuclease domain was designed to block the transcription of viral replication protein of beet severe curly top virus, 80% of transgenic *Arabidopsis* showed no symptoms against BSCTV. Similarly, the Rep gene of tomato yellow leaf curl China virus and tobacco curly shoot Yunnan virus were targeted to increase the resistance against these viruses (Yin and Qiu, 2019). Peer et al. (2015) reported the use of ZFN for the induction of targeted mutagenesis in perennial fruits including apples and fig.

The creation of lines of chickpeas with only two transgenes has been described so far (Mehrotra et al., 2011). As a consequence of the limited cloning sites inside the cassettes expressing the gene, the binary vectors employed for this transformation process have limited contribution to the transfer of more than 1-2 genes. As a result, binary vectors must be improved to transfer multiple genes in chickpeas. Dual-gene binary vectors have been created using zinc finger nucleases, which can bind and cleave lengthy DNA sequences (Zeevi et al., 2012). In chickpeas, similar procedures can be used to create a binary vector for many transgenes insertion.

Despite successful examples, various challenges are certain limitations viz; the need for DNA/protein interaction, redesigning of protein for a different DNA sequence every time is a difficult task, costly and time taking approach (Piatek et al., 2018).

#### 3.3.2 GE mediated through homing (mega) endonucleases

Site-specific restriction endonucleases can be employed to make site-directed double-strand breaks (DSBs) in the genome. Mega nucleases also known as homing endonucleases are unique enzymes with high activity and long recognition sequences (>14 bp) that digest target DNA in a site-specific manner (Epinat et al., 2003; Smith et al., 2006). Epinat et al. (2003) described the manufacture of hybrid enzymes utilizing two mega nucleases that identify new target sequences, I-Cre I and I-Dmo I. Novel mega nuclease variants that detect unique sequences with enhanced nuclease activities have also been created using specialized mutagenesis and high-throughput screening approaches (Smith et al., 2006; Arnould et al., 2007; Grizot et al., 2009).

In comparison to other SSN systems, mega nuclease has the disadvantage of being more expensive and time-demanding to develop sequence-specific enzymes for all conceivable sequences. As a result, each new genome-engineering target necessitates a first round of protein engineering to create a bespoke mega nuclease. As a result, working with mega nucleases has been difficult, and patent battles have hampered the progress (Smith et al., 2012).

#### 3.3.3 GE mediated through transcription activator like effector nucleases (TALENs)

The area of GE is rapidly expanding as new approaches and technologies emerge. GE will be required to enhance crop production since the global population is expected to reach 9.6 billion by 2050 (IPCC, 2019), while arable land shrinks. In 2009, TALEN effectors for DNA targeting were revealed. The discovery of distinctive transcription activator like effector (TALE) protein in 2011 that recognizes and activates certain plant developed through a sequence of tandem repeats led to the development of a new GE method based on chimeric nucleases dubbed TALENs (Jankele and Svoboda, 2014). TALENs are easier to construct and more widely used than ZFNs. Non-etheless, repeating sequences in the TALEN composition can enhance the probability of homologous recombination. ZFNs and TALENs are structurally and functionally identical because both of them contain the restriction endonuclease FokI.

TALE protein’s DNA binding central repeat domain is composed of a few to 33.5 repeats, each of which is made up of 34 amino acids that triggers the transcription of the target gene. Structurally, it is composed of a monomer, which binds at one specific region in the target nucleotide sequence. Monomers are found positioned at 12 and 13 repeats of 34 amino acids and are extremely variable (that are repeat variable di-residue, RVD), and are responsible for the identification of a specific nucleotide. This code degenerates and some RVDs bind to multiple nucleotides with vastly differing efficiency degrees. The targeted DNA molecule always contains the same nucleotide, that is the thymidine, before the 5′- end of a sequence, which is bound by a TALE monomer and affects the binding efficacy. The rear most tandem repeat that clips to the nucleotide at the 3′- end of the recognition site contains approximately 20 amino acid residues and is known as a half repeat (Nemudryi et al., 2014).

TALEs show high specificity towards sequence in the presence of magnesium and calcium divalent cations. However, when potassium and sodium monovalent ions are present, the TALEs are strapped to a specific as well as the non-specific region of DNA with nearly equal affinity. In comparison to monovalent ions, divalent ions in turn bind to DNA which attenuates the non-specific reciprocity between TALEs and DNA which further leads to a balanced complex (Cuculis et al., 2020).

**FIGURE 5:**
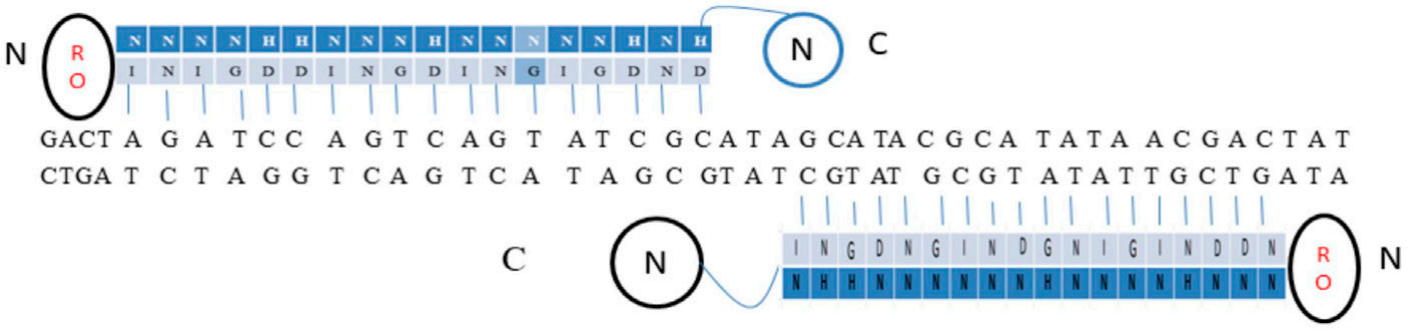
TALE activator along with a pair of TALENs.

TALENs are developed by fusing the restriction endonuclease Fok-I, a nuclease entity to a TALE DNA binding domain. To carry out precise genome editing TALEN work in pairs, binding to the DNA sequence in an opposite orientation such that the FokI domain could dimerize and cut the DNA sequence present within the spacer in between the two different binding sites. Half of the targeted sites of TALEN are conscripted in a way that the pairs are presented in an opposing intention on contradictory sides of dsDNA with an optimal sequence that acts as a spacer between them (Figure 5). In yeast, the activities of TALENs were demonstrated by combining the N- or C-terminal of TALEs with the catalytic domain of the Fok-I protein, which leads to cleavage of DNA with efficiencies equivalent to ZFN. As for as, the activity of the TALEs C-terminal domain is concerned, it is not vital. Hence, shortening the C-terminal by amino acids at +17, +28, or +63 and then fusing to the Fok-1 catalytic domain is possible that increases the efficiency too. Fok-I-based TALEN also works similarly to ZFN. Based on the length of the C-terminal TALE domain optimal spacer length is selected (Miller et al., 2011).

When the DNA-binding domains of two identical FokI nucleases come into contact, they dimerize and cut the DNA target. When these halves are created using a homodimer Fok-I, they can interact in three different ways. The left halves or right halves can combine to form a functional nuclease just as easily as the calculated interlinkage between the left and right halves of a nuclease set, which increases the likelihood that a TALEN will bind to sites with properties resembling those of the targeted DNA. Correspondingly, TALEN molecules may be linked to various parts of the genome in various combinations. It becomes more likely that a cell will be overrun by DSBs, leading to cell death and collateral loss to the DNA of surviving cells. Several obligatory heterodimer variations of FokI have been created to lessen off target toxicity. The created versions are based on mutagenesis, DNA shuffling, and structure-guided design (Joung and Sander, 2013).

This approach was created to improve genome editing efficiency, safety, and accessibility (Boch and Bonas, 2010; Urnov et al., 2010; Miller et al., 2011). The proteins imparting the effects are members of the DNA binding protein family and, like transcription factors in eukaryotic genomes, can be utilized to induce the expression of the targeted heat tolerance genes. TAL effectors (TALEs) are produced naturally by the phytopathogen *Xanthomonas oryzae* (Xanthomonas), which penetrates and reaches the nucleus of the cell and modify the transcription process to provide benefit to the pathogen (Cermak et al., 2011a). TALEs consist of a core where DNA-binding repeats are presented that regulate the binding specificity of DNA *via* an one-to-one repeated base pair binding relationship (Cermak et al., 2011a; Deng et al., 2012). TALEs can be generated to fuse any DNA sequence by modifying the number and kind of repeats (Li et al., 2013). *In vitro* and *in vivo*, fusing a TALE to nuclease results in an enzyme that is capable of creating site specific DSBs (Christian et al., 2010; Mahfouz et al., 2011; Deng et al., 2012). RVDs of the TALE repeat sequence enhance and stabilize the contact with the amino acid at the 13 positions, which give binding specificity, which are the structural foundations of TALE-DNA binding (Boch et al., 2009; Deng et al., 2012).

Because of their DNA-binding specificities, TALEs can be employed as DNA binding modules in the creation of synthetic transcriptional and epigenetic regulators. TALENs have catalysed much amusement and excitement among researchers as they can be designed easily and rapidly that ally modular DNA binding of TALE repeat domains to discrete bases in a target binding site. The primitive building blocks are used to design the domain of TALENs where DNA binds are highly conserved. Recently, co-crystal structures of TALE showed that DNA binding domains were bound to their coupled sites in the major groove of DNA (Joung and Sander, 2013).

For TALEs, several engineering platforms have been created. Furthermore, researchers examined the genetic makeup of bacteria besides, Xanthomonas and discovered that *Ralstonia solanacearum* has Ralstonia TALE-like proteins (that is RTLs) which have corresponding structure but distinct repeats with specificity as determined by numbers of RVD presence (Bogdanove et al., 2010; Remigi et al., 2011).

##### 3.3.3.1 Application of TALEN technology

The TALEN mediated genome editing approach was applied for crop enhancement for the first time in rice by disrupting the bacterial blight susceptibility gene Os SWEET14 and producing a mutant rice to show resistance towards bacterial blight (Li et al., 2012). TALENs have also been utilized to knock out three TaMLO homeologs in wheat to develop powdery mildew resistant wheat (Wang et al., 2014). Char et al. (2015) generated mutants of maize with the glossy phenotype, reduced amount of epicuticular wax in the leaves, and the ability to be surface manured by eliminating the maize GL2 gene. TALEN mediated mutagenesis has increased the composition of the cell wall and saccharification effectiveness in sugarcane (Jung and Altpeter, 2016; Kannan et al., 2018). During cold storage, product quality declines majorly because of the accumulation of reducing sugars. As observed in potato tubers, knocking down the vacuolar invertase (VInv) gene resulted in tubers with undetectable amounts of harmful reducing sugars (Clasen et al., 2016). Integrating TALENs and donor DNA in Gemini virus replicons markedly escalate the copy number and homologous recombination efficiency *via* introducing a powerful promoter upstream of the gene regulating anthocyanin biosynthesis resulting in purple tomatoes with an increased amount of anthocyanin (Čermák et al., 2015). Recently, one of the mitochondrial *orf* genes, *orf 312* (CMS-associated gene), knocked out by this approach showed that it is responsible for pollen abortion and leads to cytoplasmic male sterility in rice (Takatsuka et al., 2022). These examples show how TALEN technology can be used to improve crops including chickpea heat tolerance and yield traits in a variety of ways. However, the production of TALE repeats remains a difficult path to follow and harness the efficacy of gene targeting.

#### 3.3.4 GE mediated through Clustered Regularly Interspaced Short Palindromic Repeats (CRISPR)

**FIGURE 6:**
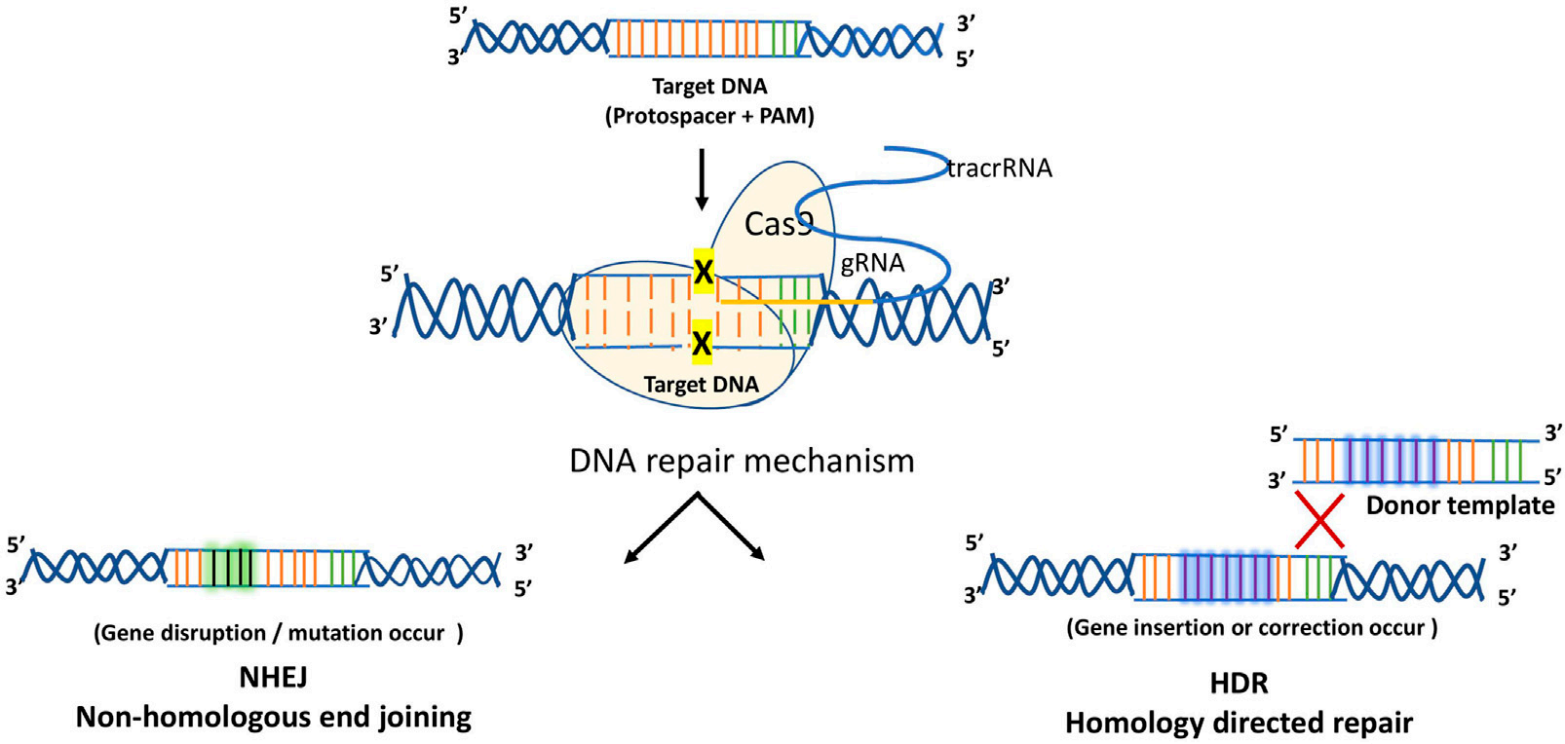
Generalized mechanism of CRISPR/Cas-9.

CRISPR technology was introduced 2 years later, after the discovery of the TALEN proteins. CRISPR, which consists of non-coding RNAs and Cas proteins, was developed and has since become widely employed. Unlike first generation genome editing approaches, CRISPR/Cas9 is easy to design, clone, and the similar Cas9 protein theoretically can be used with a variety of guide RNAs to target several locations throughout the genome. The most commonly used genome editing tools are TALENs and CRISPR associated Cas9. Each represents a type of engineered nuclease that can be customized to recognize, bind, and cleave a specific sequence in the genome. TALENs are entirely protein-based, and CRISPR/Cas9 has both protein and RNA components (Musunuru, 2017). Unlike the chimeric TALEN proteins, the CRISPR/Cas9 system recognizes the DNA site which needs to be altered by a complementary interaction between a non-coding RNA and the targeted site. Hence, it leads to the formation of a complex consisting of non-coding RNA and Cas9 proteins having nuclease activity. The generalized mechanism of CRISPR technology is depicted below as Figure 6.

CRISPR associated Cas9 system, is the most prominent and innovative genome editing approach which has recently become popular. CRISPR/CAS-9 has been widely accepted due to its preciseness, high efficiency, and utility to ameliorate abiotic and biotic stress tolerance in plants as detailed mentioned in Table 2. CRISPR is palindromic repeat sequences found in the bacterial genome separated by a spacer of 32–36 base pairs. There are several CRISPR/Cas9 systems but primarily classified into three types; type I, II, and III. For plant genome editing, CRISPR/Ca9 type II is frequently used. It is an adaptation of the Gram-positive *Streptococcus pyogenes* system (Le Rhun et al., 2019). Presently, it has been believed to be an efficient and precise *in vitro* as well as *in vivo* genome editing tool and many tailored Cas9 complexes have been utilized to increase the frequency of selectivity of target and reduce the chances of off target cleavage after proof-of-concept demonstrations by core CRISPR/Cas9 module (viz- Nmcas9, Sacas9, and Stcas9) in plants. Additionally, utilization of Cas9 enzymes from different bacterial strains have increased the specificity and efficacy of gene editing procedures as presented in Table 3 (Jaganathan et al., 2018).

**TABLE 2:** Application of CRISPR based genome editing approach in plants for biotic, abiotic, and nutritional traits.

Crop	Method	Target gene	Stress/trait	References
Biotic stress				
A. thaliana/	NHEJ	dsDNA of virus (A7, B7, and C3	Beet severe curly top virus resistance	Ji et al. (2014)
Rice	NHEJ	OsERF922 (ethylene responsive	Blast Resistance	Wang et al. (2016)
Bread wheat	NHEJ	TaMLO-A1, TaMLO-B1, and	Powdery mildew resistance	Wang et al. (2014)
Cucumber	NHEJ	eIF4E (eukaryotic translation		Chandrasekaran et al. (2016)
Abiotic stress				
Maize	HDR	ARGOS8	Increased grain yield under drought stress	Shi et al. (2017)
Tomato	NHEJ	SlMAPK3	Drought tolerance	Wang et al. (2017)
A. thaliana	NHEJ	UGT79B2, UGT79B3	Susceptibility to cold, salt, and drought stresses	Zhao et al. (2016)
Rice	HDR, NHEJ	OsPDS, OsMPK2, OsBADH2	Involved in various abiotic stress tolerance	Xie and Yang (2013)
Rice	NHEJ, HDR	OsMPK2, OsDEP1	Yield under stress	Shan et al. (2014)
Nutritional Traits				
Rice	NHEJ	25604 gRNA for 12802 genes	Creating genome wide mutant library	Meng et al. (2017)
Maize	NHEJ	ZmIPK1A ZmIPK andZmMRP4	Phytic acid synthesis	Liang et al. (2014)
Wheat	HDR	TaVIT2	Fe content	Connorton et al., 2017
Soybean	NHEJ	GmPDS11 and GmPDS18	Carotenoid biosynthesis	Du et al. (2016)
Tomato	NHEJ	Rin	Fruit ripening	Ito et al. (2015)
Potato	HDR	ALS1	Herbicide resistance	Butler et al. (2016)
Cassava	NHEJ	MePDS	Carotenoid biosynthesis	Odipio et al. (2017)

**TABLE 3:** Summary of CRISPR-Cas enzymes.

Class	Type	Subtype	Effector	Target	Nuclease domains	TracrRNA requirement	PAM/PFS
1 (Multi-Cas proteins)	I	A,B,C,D,E,F,U	Cascade	dsDNA	HD fused to Cas3	No	-
1 (Multi-Cas proteins)	III	A,B,C,D	Cascade	ssRNA	HD fused to Cas10	No	–
1 (Multi-Cas proteins)	III	A,B,C,D	Cascade	ssRNA	HD fused to Cas10	No	–
1 (Multi-Cas proteins)	IV	A, B	Cascade	dsDNA	unknown	No	–
2(Single-Cas protein)	II	A	SpCas9	dsDNA	RuvC, HNH	Yes	NGG
2 (Single-Cas protein)	II	A	SaCas9	dsDNA	RuvC, HNH	Yes	NNGRRT
2(Single-Cas protein)	II	B	FnCas9	dsDNA/ssRNA	RuvC, HNH	Yes	NGG
2(Single-Cas protein)	II	C	NmCas9	dsDNA	RuvC, HNH	Yes	NNNNGATT
2(Single-Cas protein)	V	A	Cas12a (Cpf1)	dsDNA	RuvC, Nuc	No	5° AT-rich PAM
2(Single-Cas protein)	V	B	Cas12b (C2c1)	dsDNA	RuvC	Yes	5° AT-rich PAM
2(Single-Cas protein)	V	C	Cas12c (C2c3)	dsDNA	RuvC	Yes	5° AT-rich PAM
2(Single-Cas protein)	VI	A	Cas13a (C2c2)	ssRNA	2xHEPN	No	3° PFS: non-G
2(Single-Cas protein)	VI	B	Cas13b (C2c4)	ssRNA	2xHEPN	No	5° PFS: non-C; 3° PFS:NAN/NNA
2(Single-Cas protein)	VI	C	Cas13c (C2c7)	ssRNA	2xHEPN	No	–
2(Single-Cas protein)	VI	D	Cas13d	ssRNA	2xHEPN	No	–

##### 3.3.4.1 Applications of CRISPR/Cas9 systems

CRISPR can make deliberate changes in genome structures hence it has a tremendous impact on bioengineering and molecular biology. The technology was used to improve the colour, shelf life, and commercial attractiveness of fruits and vegetables by reducing the amount of toxic steroidal glycoalkaloids. A boost in amylose, starch, aroma, good fats like oleic acid, etc., and a decrease in gluten proteins and unsaturated fatty acid content and so on were among the other modifications (Jiang et al., 2017; Sun et al., 2017). Thus, in crop plants, the CRISPR/cas9 technique can be exploited to improve the yield and quality by increasing the shell life, amending colour, size, texture, etc. (Xing et al., 2020).

To develop biotic resistant crops an attempt was made, where initiation factor elF4E of cucumber was inactivated using CRISPR/Cas9 system, resulting in plants found to be resistant towards cucumber vein yellowing virus. Similarly, grape knockout of VvWRKY52 increased tolerance against fungal infection. In another experiment conducted on rice, CRISPR/Cas9 knocked out the LAZY1 gene resulting in a tiller-spreading phenotype that may boost yield in a certain environment (Miao et al., 2013; Li et al., 2016). In another study, three different genes including Grain Number 1a (Gn1a), dense and erect panicle (dep1), and grain size (GS3) of the rice cultivar Zhonghua 11 were mutated by the CRISPR/Cas9 system those showed a greater number of grains with an increase in size and dense erect panicles. Recently, the role of *Oryza sativa* senescence associated protein during drought has been explored by editing drought induced genes (Park et al., 2022).

Chickpea production is hampered by drought, low and high temperatures, and other abiotic conditions (Gaur et al., 2008; Mantri et al., 2010; Jha et al., 2014). Recently, two potential genes, 4 coumarate ligase (4CL) playing important role in phenylpropanoid metabolism, and Reveille 7 (RVE7) involved in circadian rhythm were chosen for CRISPR/Cas9 editing in chickpea protoplast, both of which are linked to drought tolerance. The 4CL enzyme is engaged in the phenylpropanoid metabolism pathway during the production of lignin. To knock off these targeted genes in chickpeas, researchers used DNA free CRISPR/Cas9 editing tool. In chickpeas, protoplast editing is a revolutionary technique for accomplishing targeted mutagenesis. In comparison to the 4CL gene, the RVE7 gene showed excellent *in vivo* editing effectiveness. According to Ninan et al. (2019), in the leaves of chickpeas, cytokines have increased sink activities. Isopentenyl transferase controls the earliest steps in the synthesis of cytokines (IPT). The cytokinin dehydrogenase or oxidase is now in charge of controlling cytokinin breakdown. Root-specific promoter CaWRKY31 of chickpeas could be used to explore the mechanism behind how cytokinin diminution impacts the development of root architecture and tolerance towards drought. In Arabidopsis and chickpeas to study definite and indeterminate growth patterns, a root specific promoter CaWRKY31 can be used. In the model plant Arabidopsis and chickpea, it is observed that root-specific CaCKX6 expression increased the proliferation of lateral roots plant biomass without impairing the vegetative and reproductive development. Root cytokinin oxidase/dehydrogenase (CKX) gene activity was seen to be increased in transgenic chickpea strains. CKX gene functional characterization studies in chickpeas have only recently begun. Gene editing tools such as TALENs and CRISPR/Cas9 approach can be quite useful in this situation (Mahto et al., 2022). Gene editing technologies can help with knock-ins in addition to knockouts.

Heat, drought, floods, temperature extremes, salt, heavy metals, radiation, and other factors can contribute to abiotic stress. Stress has a significant impact on the yield of crops. Several crops have been mutated to defend against abiotic (Shan et al., 2013; Klap et al., 2017). To boost drought tolerance in maize, researchers employed CRISPR/Cas9 to introduce a promoter at a specific region (Shi et al., 2017). Site specific genomic change has previously been accomplished using gene editing tools like zinc finger nuclease and transcription activator like effector nucleases, but these tools have limitations (Gupta and Musunuru, 2014).

Biotic stress, on the other hand, is caused by microbes like fungi, bacteria, and viruses. Several crops have been mutated to defend against biotic stresses (Lu et al., 2018). Hybrid breeding, which includes improvements in hybrid wheat seed production, is another approach to increasing crop output. Hybrid crops are effective high yielding cultivars today, yet hybrid seed production requires emasculation to avoid self-pollination.

These gene editing technologies like TALENs or CRISPR/Cas9 can be quite useful in the creation of non-genetically modified crops that have the desired trait, boosting yield potential under biotic and abiotic stress situations (Mahto RK et al., 2022).

**FIGURE 7:**
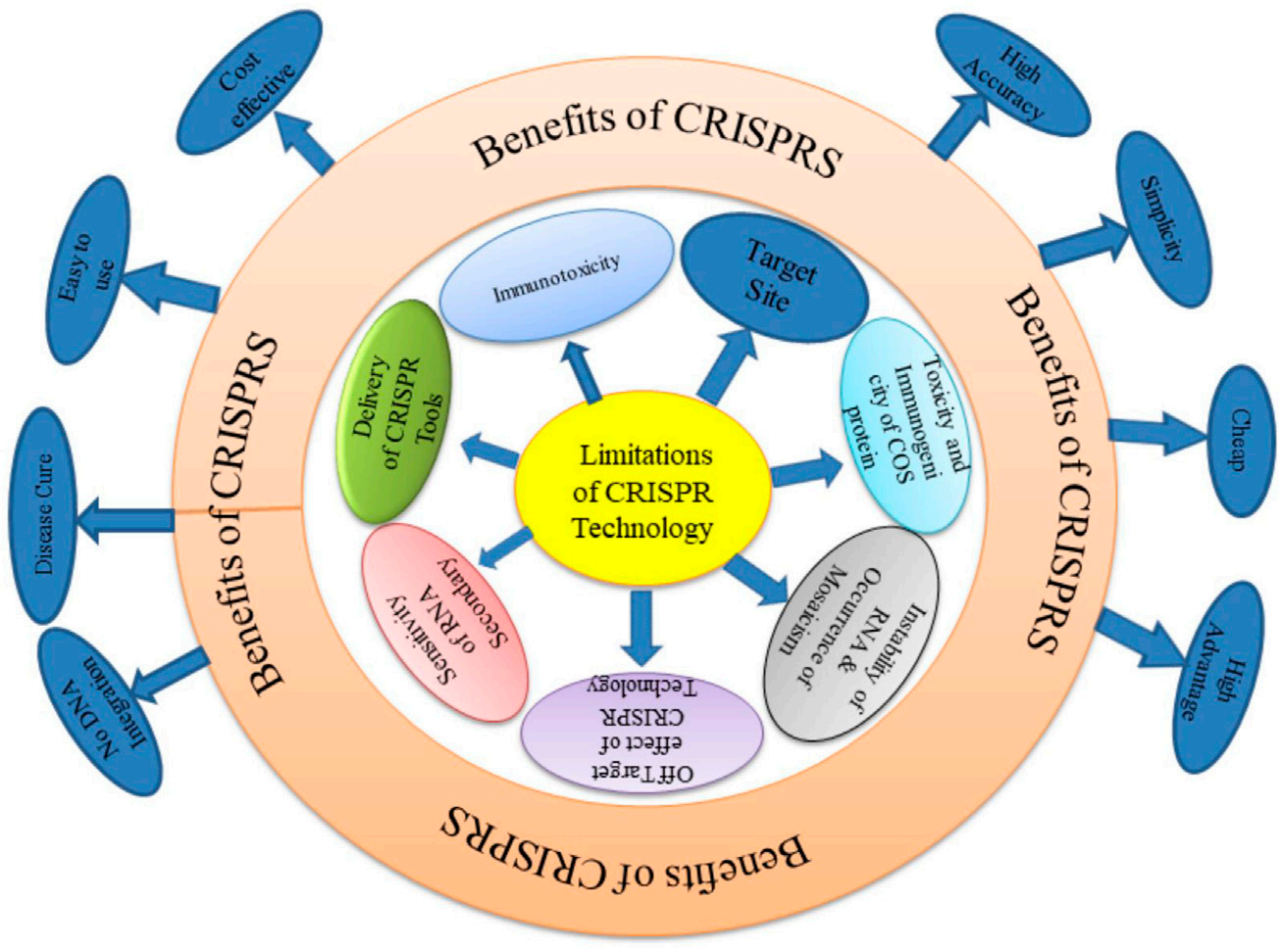
Limitations and benefits of CRISPR technology.

However, a major drawback of CRISPR technology compared to other genome editing tools is the high frequency of off target mutations even to the extent of up to 50%. (Zhang et al., 2015). The most difficult problem so far has been getting the CRISPR system into the target cells. Each crop including chickpea that uses CRISPR/Cas9 has intrinsic restrictions. At first, it is impossible to determine potential editing targets of interest or evaluate gRNAs off target behaviour without access to or incomplete assembly of a genome sequence (Hahn and Nekrasov, 2019). There is a need for additional research in this field due to technical challenges in creating viable transgenic chickpeas and the lack of a stable transient system of expression for quick study of gene expression and function (Badhan et al., 2021). The generalized limitations and benefits of CRISPR technology is depicted below as Figure 7.

### 3.4 GE mediated through base and prime advanced approaches

Over the past few years, numerous prime editing (PE) and base editing (BE) variants have been created and experimentally validated in plants (Molla et al., 2021). These are two recently established genome engineering techniques that can rapidly insert specific modifications into target regions without the use of donor DNA templates or DSB creations. Applications like controlling cis-elements, altering RNA splice sites, including synthetic miRNAs, or customizing miRNA binding sites are made possible using PE and BE technologies. The binding locations of effectors produced by fungal infections to target plant susceptibility genes may also be altered by these methods and heritable resistance may be passed down in this manner (Van Vu et al., 2022). Both base editing and prime editing have been tested on a variety of plant types and proven to be effective.

#### 3.4.1 Base editing

**FIGURE 8:**
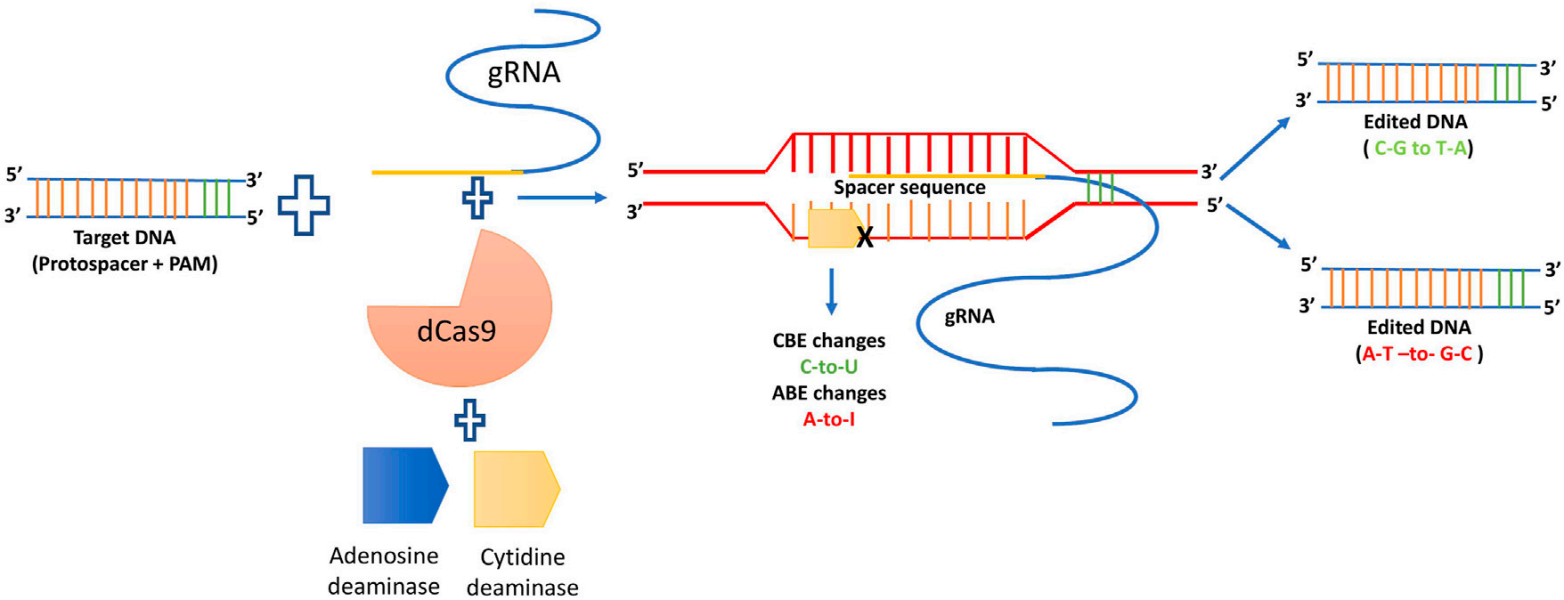
Generalized mechanism of base editing.

BE is a game changing method for precisely implanting point mutations at the appropriate places without the use of donor DNA templates or the production of double strand breaks (Rees and Liu, 2018). First cytosine base editor (CBE) was produced using a SpCas9 (D10A) nickase in combination with a cytidine deaminase and an uracil glycosylase inhibitor (UGI) to make the transition from C G to T A (Komor et al., 2016). Following that cytidine deaminase will deaminate the exposed non-target DNA strand changing cytosine (C) to uracil (U) resulting in a C to T base change during DNA repair and replication. Structurally, the adenine base editor (ABE) is analogous to the CBE, and using *E. coli* transfer RNA adenosine deaminase (ecTadA), it converts adenine A) to inosine (I) in the non-target strand (Gaudelli et al., 2017). Moreover, in a variety of plant species, CBEs and ABEs have been employed to research the function of genes undiscovered and improve crop qualities (Molla and Yang, 2019; Mishra et al., 2020). To handle CT and AG conversion in a genomic area of interest, an interesting approach was applied, and a dual base kind of editor was constructed by fusing cytidine and adenosine deaminases into Cas protein. Discretely, CBE, ABE as well as dual base editors, have a similar mode of action: deamination of C and A by cytidine and adenosine deaminase, respectively (Abdallah et al., 2021). The generalized mechanism of base editing technology is depicted below as Figure 8.

Although CRISPR based precision genome editing technologies have evolved and flourished fast, these tools have been unable to reach organelle genomes because of the non-availability of guide RNA as well as Cas proteins inside organelles. Hence, it is important and needs to explore the possible ways to approach organelle specific gene editing of monocots and dicots to decipher the function of the gene and limit the off targets’ chance. However, very recently, organellar genome engineering has been described (Mok et al., 2020) and the group has discovered the deaminase domain of the bacterial toxin DddA which is structurally similar to that of APOBEC enzymes and deaminates the cytosines in double stranded DNA (dsDNA). DddAtox is being integrated with organelle focused transcription activator like effector (TALE) repeat arrays, which directly deaminates dsDNA in organellar genomes. Despite the efficiency of DdCBEs in a variety of species of the plants, various issues such as DddAtox deaminase sequence preference and likely editing of off target sites must be directed before precise organellar genome editing in plants can be carried out (Azameti and Dauda, 2021).

#### 3.4.2 Prime editing

**FIGURE 9:**
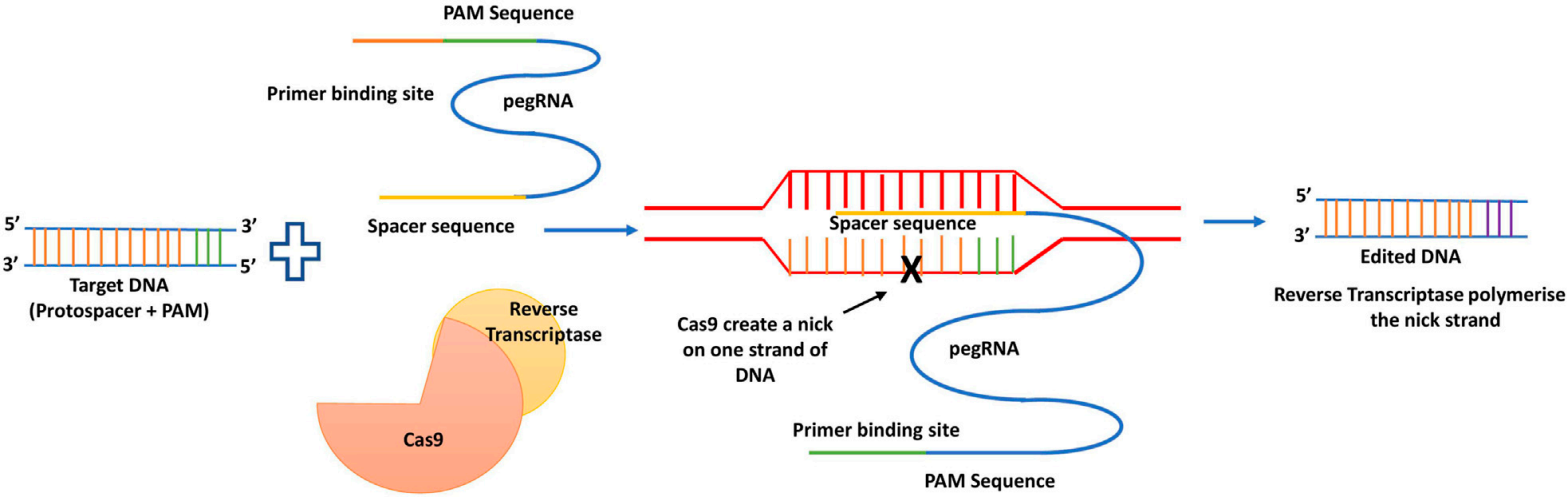
Mechanism of prime editing.

PE is a non DSB genome editing method that results in all feasible base conversions, tiny indels, and combinations of them at selected regions (Anzalone et al., 2019). The target site is specified using guide RNA with a 5′spacer sequence. The Cas9 nickase reverse transcriptase and fusion proteins are the prerequisites. The prime editing guide RNA called pegRNA, which guides the fusion of proteins to identify the target site before causing a nick on the non-target strand, after which it anneals with primer binding site (PBS) and finally primes the reverse transcriptase of the reverse transcriptase template, which then copies the right sequence into the target after a lengthy DNA repair mechanism (Anzalone et al., 2019). The generalized mechanism of prime editing technology is depicted below as Figure 9.

The PE method has been used with a variety of plants (Xu R. et al., 2020; Butt et al., 2020; Xu W. et al., 2020; Hua et al., 2020; Jiang et al., 2020; Li et al., 2020). In comparison to mammalian cells editing frequencies are lower in monocot plants and in dicot species not at all (Lu et al., 2021; Wang et al., 2021). PE events have been observed in stable transgenic lines of two important crops *Oryza sativ*a and *Solanum Lycopersicon*, however, the ratio of homozygous in comparison to biallelic edits is significantly low (Xu R. et al., 2020; Xu W. et al., 2020; Hua et al., 2020; Li et al., 2020; Lin et al., 2020; Lu et al., 2021), indicating PE’s inefficiency in plants (Hua et al., 2020). Further, Biswas et al. (2022) have shown a low range of prime editing efficiency in legumes, ranging from 0.2% to 0.5% of protoplast cells showing the targeted edits, a higher editing efficiency is expected once transgenic plants are developed. However, further optimization of the prime editing system should improve editing efficiency in legumes including chickpea.

##### 3.4.2.1 Application of base editors and prime editors

Research articles related to DSB independent genome editing tools, base editing, and prime editing considered them to be more predictive than DSB dependent genome editing tools, which have various advantages including knowing about the function of gene and precision crop breeding (Komor et al., 2016; Gaudelli et al., 2017; Anzalone et al., 2019). Bes, PEs can interrupt genes by incorporating stop codons, alternately inactivating, splicing sites, which are highly conserved in coding regions of genes for thwarting undesired mutations in the genome, synthesis of aberrant proteins, (Billon et al., 2017; Kluesner et al., 2021; Ren et al., 2021). In addition, BEs and PEs can precisely alter possible gene regulatory regions including sites where miRNA or transcription factors bind or modifies post transitional regions and can act on the open reading frame to infer their activities (Xing et al., 2020; Ren et al., 2021).

## 4 Conclusion and future perspective

The crop genome engineering inclusive of genomics and genome editing tools have already been successfully employed in several crops, although it is still in its early phase for production enhancement and abiotic stresses including heat tolerance, drought, salinity, etc in chickpea. Various genomic approaches viz; multi-omics, transcriptomics, proteomics, metabolomics, pan and genome editing technologies have tremendous potentials to influence the plant breeding techniques to guard crop plants against numerous abiotic/biotic stresses and augment crop yield. Editing the target DNA sequence by adding, deleting, or substituting nucleotide bases are cutting edge molecular biology techniques and Genome amending procedures viz; SSRs, ODMs, SSNs inclusive of ZFNs, TALENs, Mega nucleases, CRISPR/Cas9 and advanced approaches viz; Base Editors, Primer Editors are used. The CRISPR/Cas9 technologies corroborate the utmost operational GE machinery since these are precise, less expensive, speedy, and consent for numerous site-specific genome editing. SSNs have been utilized to elucidate the activities of many essential genes in plants that could be exploited to boost agricultural yield and often SSN induced NHEJ were used in polyploidy plants to investigate gene function and trait development which resulted in gene deletions. Recently, scientists are focusing on fabricating plant genomes to make them withstand climatic changes. In defiance of its success in the laboratory, gene editing technology for climate change has yet to demonstrate a significant impact in the real world as regulations, societal hurdles, and proscriptive policies, among other externalities outside the technical limits stated have hampered the adoption of these technological advancements. However, current technical advances are rapidly expanding and thanks go to the continued efforts of both public and commercial organizations. Genetic engineering approaches as mentioned above that alter minimal DNA/chromatin configurations, but exact modifications in the genome or precise insertion of small DNA fragments are attractive possibilities for worldwide regulatory overhaul, policy improvements, and increased consumer acceptance. Naturally, the advantages of gene editing applications will only be recognized once farmers and producers have access to these revolutionary technologies. Despite technological restrictions, socio-political barriers must overcome and gene-modified products should be widely adopted. Thus, CRISPR gene editing tool is an essential forward step for agricultural adaptability in the face of negative climate impact and holds the great possibilities for harnessing the betterment of future agriculture including chickpea enhanced capabilities for cytokinin dehydrogenase, nitrate reductase, superoxide dismutase to induce drought resistance, heat tolerance and higher yield higher yield to encounter global climate change, hunger and nutritional threats.
